# MicroRNA-638 inhibits the progression of breast cancer through targeting HOXA9 and suppressing Wnt/β-cadherin pathway

**DOI:** 10.1186/s12957-021-02363-7

**Published:** 2021-08-20

**Authors:** Qian Xu, Qianqian Zhang, Mengli Dong, Yuan Yu

**Affiliations:** Department of Oncology, Weifang People’s Hospital, No. 151, Guangwen Street, Kuiwen District, Weifang, 261041 People’s Republic of China

**Keywords:** miR-638, HOXA9, Breast cancer, Wnt/β-cadherin pathway

## Abstract

**Background:**

Previous studies had shown that microRNA-638 (miR-638) exhibited different effects in malignant tumors. Moreover, the function of miR-638 has not been reported in breast cancer. Hence, we designed this research to explore the function of miR-638 in breast cancer.

**Methods:**

Firstly, miR-638 expressions were measured in breast cancer tissues via RT-qPCR. Protein expressions were detected through immunocytochemical (IHC) assay and western blot analysis. Then, Cell Counting Kit-8 (CCK-8) assay and Transwell assay were conducted to observe proliferation and motility of the cells. Dual luciferase assay was performed to confirm the binding site between miR-638 and Homeobox protein Hox-A9 (HOXA9).

**Results:**

Reduced expression of miR-638 was detected in breast cancer. And low miR-638 expression was related to poor prognosis in patients with breast cancer. Functionally, the viability, migration, and invasion of the breast cancer cells were suppressed by miR-638 overexpression. Furthermore, miR-638 can directly bind to HOXA9, and increased expression of HOXA9 was also detected in breast cancer. In particular, HOXA9 upregulation can impair anti-tumor effect of miR-638 in breast cancer, and miR-638 can hinder the Wnt/β-cadherin pathway and epithelial-mesenchymal transition (EMT) in breast cancer.

**Conclusion:**

miR-638 inhibits breast cancer progression through binding to HOXA9.

## Introduction

Breast cancer usually occurs in the epithelium of the breast gland that seriously endangers women’s lives [1]. Breast cancer usually occurs in women, and only about 1-2% of the patients with breast cancer are men [2]. Breast cancer accounts for 7-10% of human malignant tumors, second only to cervical cancer in women [3]. Among them, the incidence of postmenopausal women ranging from 40 to 60 years old is higher [3]. Similar to a few types of tumors, such as thyroid cancer, the natural development of breast cancer is usually long, and it takes 7 to 8 years for the tumor to reach a sphere with a diameter of 1 cm [4]. Moreover, surgical treatment is the main therapy for breast cancer, and radiotherapy is one of the local treatment methods [5]. Prior to tumor metastasis, surgery and radiation alone can cure the vast majority of breast cancer patients. Once metastasis occurs, routine treatment can only cure a small number of breast cancer patients [6]. In addition, there are many factors influencing the prognosis of breast cancer patients, among which tumor invasion scope and pathological and biological characteristics are the main ones [7]. And the best way to reduce the mortality of breast cancer is early diagnosis and early treatment.

MicroRNAs (miRNAs) have been reported to affect the expressions of their targets through binding with their 3′-UTR. Besides, miRNAs are widely recognized as powerful regulators which involve many processes of breast cancer. For instance, miR-384 was found to inhibit breast cancer progression by targeting ACVR1 [8]. Inversely, miR-374a promoted tumor metastasis and progression by downregulating LACTB and predicts unfavorable prognosis in breast cancer [9]. In addition, it was reported that miR-17-5p could serve as a novel predictor for breast cancer recurrence [10]. Recently, previous studies showed that miR-638 was an important regulator for the occurrence and development of human cancers. For instance, miR-638 was found to restrain the tumorigenesis of gastric cancer [11]. On the contrary, miR-638 was found to promote cell metastasis and inhibit apoptosis in melanoma [12]. miR-638 can also predict the outcome of the patients with non-small cell lung cancer after chemotherapy [13]. Moreover, Zhao et al. found that potentiation of docetaxel sensitivity was regulated by miR-638 via regulation of STARD10 pathway in human breast cancer cells [14]. However, the function of miR-638 in breast cancer remains unclear.

Besides that, it has been found that the activation of Wnt/β-catenin pathway can promote the progression of human cancers [15]. β-catenin belonging to the Wnt/β-catenin signaling pathway was reported to participate in tumorigenesis [16]. Wnt/β-catenin signaling pathways was also identified as therapeutic targets in cancer [17]. Moreover, it was demonstrated that the dysfunction of Wnt and Notch signaling pathways regulated the progression of breast cancer [18]. The activation of oncogene HOXA9 was demonstrated to associate with the prognosis of human glioblastoma [19]. Brumatti G et al. demonstrated that HOXA9 could mediate the survival of myeloid progenitors [20]. HOXA9 had been found to modulate human breast tumor phenotype [21]. In addition, it was found that Wnt/β-catenin pathway was associated with HOXA2 in mouse embryo [22]. However, the functions of Wnt/β-catenin pathway and HOXA9 were not reported in breast cancer.

Moreover, epithelial to mesenchymal transition (EMT) had a great effect on human cancer cell metastasis [23]. Han et al. proposed that miR-30d could mediate breast cancer invasion, migration, and EMT by targeting KLF11 [24]. But the relationship between miR-638 and EMT is still elusive in breast cancer. Therefore, this study attempted to investigate the role of miR-638 in breast cancer, and illustrate the regulation mechanism of miR-638 on breast cancer.

## Materials and methods

### Experimental samples

The experimental tissues were acquired from fifty-two patients with breast cancer in CaoXian People’s Hospital. These patients provided informed consents and only received surgery before this study. The tissues were frozen in liquid nitrogen and stored in a −80 °C refrigerator. This research was approved by the Institutional Ethics Committee of CaoXian People’s Hospital.

### Cell lines culture

The MDA-MB-468, MDA-MB-231, MCF-7 cell lines, and human breast epithelial cell line MCF10A were purchased from ATCC (Manassas, VA). These cell lines were then cultured in DMEM medium with 10% fetal bovine serum (FBS) at 37 °C with 5% CO_2_.

### Cell transfection

miR-638 mimics or inhibitor and negative control (NC, GenePharma, Shanghai, China) were respectively transfected into MCF-7 cells with Lipofectamine 2000 (Invitrogen, Carlsbad, USA).

### RT-qPCR analysis

TRIzol reagent (Invitrogen, Carlsbad, USA) was applied to extract total RNA. The reverse transcription of cDNA was performed by PrimeScript reverse transcription kit (Qiagen, Valencia, USA). RT-qPCR was conducted using SYBR green Supermix (Bio-Rad, Richmond, USA) on ABI 7500 Fast system (Applied Biosystems, CA, USA). The internal control for miR-638 or HOXA9 is U6 or GAPDH. And the mRNA expression was calculated by the 2^−△△ct^ method.

### CCK-8 assay

The transfected cells (4 × 10^4^) were put in 96-well plates and incubated for 0, 24, 48, and 72 h in an incubator at 37 °C with 5% CO_2_. Next, each well was added with 10 μl CCK-8 reagents for 2 h (Dojindo, Tokyo, Japan). Finally, the absorbance of each well at 450 nm was detected by a microplate reader (Molecular Devices).

### Transwell assay

Cell migration and invasion were detected using Transwell chambers (8-μm pore size membranes). Matrigel (BD Biosciences, USA) was added in the upper surface for observing cellular invasion. The lower chamber was added with 10% FBS. MCF-7 cells with miR-638 mimic or inhibitor were incubated in the upper chamber with serum-free medium for 48 h at 37 °C with 5% CO_2_. The moving cells were fixed with methanol and stained with crystal violet. Then, the number of removed cells was observed using a microscope.

### Dual luciferase assay

The psiCHECK-2 vector (Promega, Madison, USA) containing the 3′-UTR of wild type or mutant HOXA9 was constructed. Then, the whole vector and miR-638 mimics were transfected into MCF-7 cells. The luciferase activity was detected using dual luciferase assay system (Promega, USA).

### Immunohistochemistry (IHC)

After dewaxing, hydrating, and washing the ovary tissues section, the cells were blocked with 5% goat serum (diluted in PBS). Next, the cells were incubated with anti-HOXA9 (nucleus) antibody at 37 °C for 1-2 h. Then, the cells were incubated with appropriate secondary antibodies at 37 °C for 1 h. After washing, the color development of this section was performed using DAB mixture. Next, we washed, counterstained, dehydrated, transparentized, and mounted the section. Microscope was used to capture images.

### Western blot analysis

The protein samples were obtained using RIPA lysis buffer. The protein was separated by 10% SDS-PAGE. After blocking with 5% skim milk, the protein was transferred in PVDF membranes. The membranes were then incubated with E-cadherin, N-cadherin, vimentin, β-cadherin, p-β-cadherin, HOXA9, and GAPDH antibodies overnight at 4°C. The protein was then incubated with secondary antibodies for 2 h at room temperature. Finally, protein expression was detected by Electro-Chemi-Luminescence system (ECL, Pierce Biotechnology, USA).

### Statistical analysis

Data are analyzed using SPSS 19.0 or Graphpad Prism 6. Chi-squared test was adopted to analyze the relationship between HOXA9 and clinical features in breast cancer patients. Tukey’s one-way ANOVA was used to calculate the difference between multiple groups. The survival differences were compared by Kaplan-Meier analysis (log-rank test). *P* < 0.05 indicates significant difference.

## Results

### miR-638 expression was decreased in breast cancer tissues

**Fig. 1:**
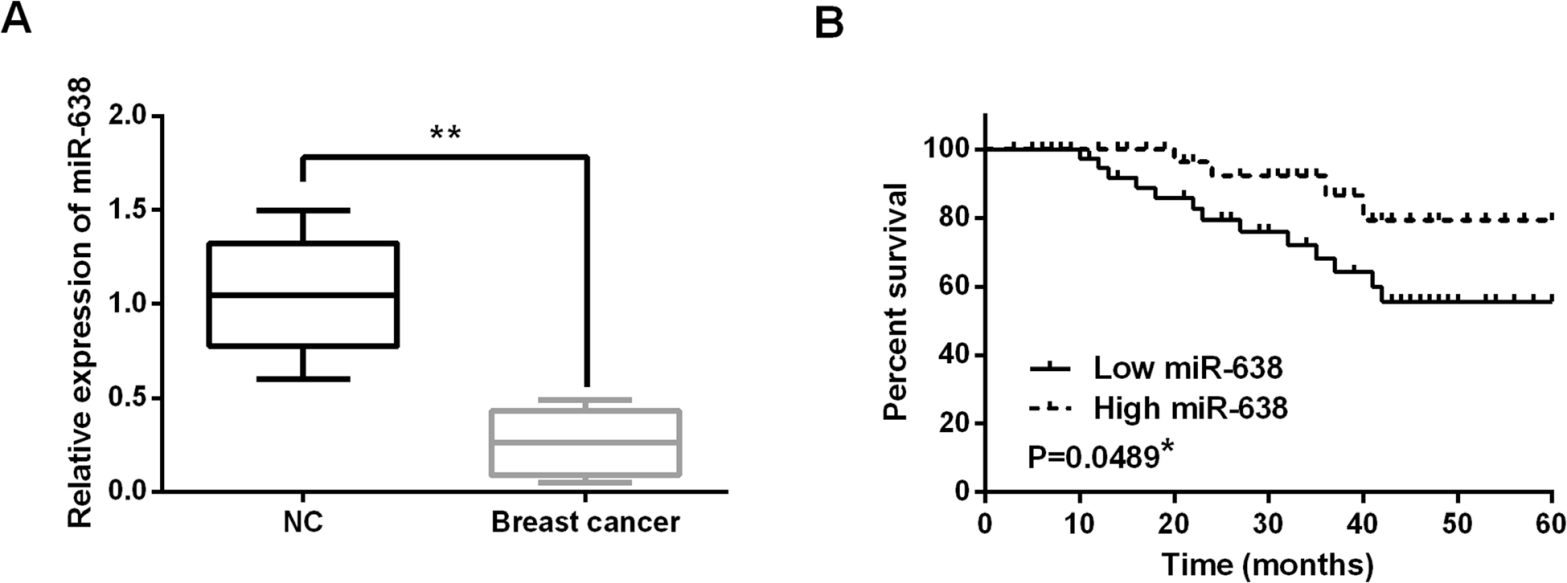
miR-638 expression was decreased in breast cancer tissues. (**A**) The expressions of miR-638 in breast cancer tissues. (**B**) High miR-638 expression was correlated with longer overall survival of breast cancer patients. *P < 0.05, **P < 0.01

Firstly, miR-638 expressions were observed in breast cancer tissues. Compared to normal tissues, miR-638 expressions were downregulated in breast cancer tissues (Fig. 1A). Moreover, miR-638 was found to be associated with tumor size, TNM stage, and lymph node metastasis (Table 1). Furthermore, low miR-638 expression predicted unfavorable prognosis in breast cancer patients (Fig. 1B). The results suggested that abnormal miR-638 expression was related to the tumorigenesis and prognosis of breast cancer.

**Table 1:** Relationship between miR-638 expression and their clinic-pathological characteristics of breast cancer patients

Characteristics	Cases	miR-638		*P* value
		High	Low	
**Age (years)**				0.11
≥ 50	27	12	15	
< 50	25	10	15	
**Tumor size**				0.022*
< 3 cm	35	11	24	
≥ 3 cm	17	5	12	
**ER status**				0.256
Negative	22	8	14	
Positive	30	13	17	
**PR status**				0.119
Negative	24	10	14	
Positive	28	13	15	
**HER2 status**				0.391
Negative	32	14	18	
Positive	20	7	13	
**Lymph node metastasis**				0.004*
Yes	37	12	25	
No	15	5	10	
**TNM stage**				0.049*
I-II	14	6	8	
III-IV	38	15	23	

Statistical analyses were performed by the *χ*^2^ test

*P < 0.05 was considered significant

### Overexpression of miR-638 inhibited breast cancer progression

**Fig. 2:**
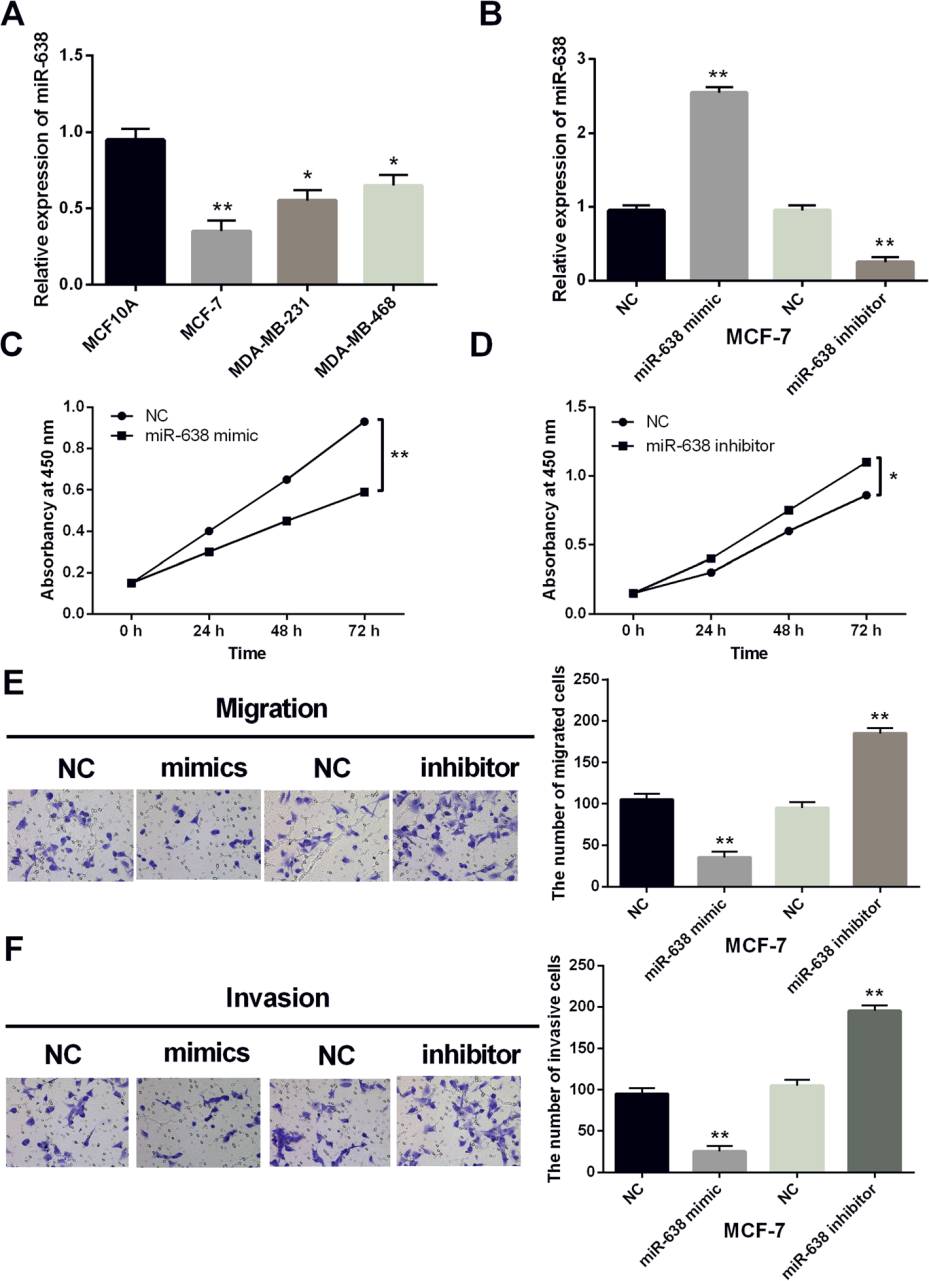
Overexpression of miR-638 inhibited the progression of breast cancer. (**A**) The miR-638 expression in MCF-7, MDA-MB-231, MDA-MB-468, and MCF10A cell lines. (**B**) The expression of miR-638 was examined in MCF-7 cells with miR-638 mimics or inhibitor. (**C**, **D**) The cell proliferation was measured in cells containing miR-638 mimics or inhibitor. (**E**, **F**) Cell migration and invasion analysis in cells containing miR-638 mimics or inhibitor *P <0.05, **P <0.01

Then, miR-638 expressions were detected in MDA-MB-468, MDA-MB-231, MCF-7, and MCF10A cell lines. Similarly, downregulation of miR-638 was also identified in MDA-MB-468, MDA-MB-231, and MCF-7 cell lines in contrast to MCF10A cells (Fig. 2A). Next, miR-638 mimics or inhibitors were transfected into MCF-7 cells to detect its role in breast cancer. The transfection efficiency was detected by RT-qPCR (Fig. 2B). Functionally, miR-638 overexpression inhibited cell proliferation in breast cancer (Fig. 2C). On the contrary, the knockout of miR-638 promoted the proliferation of MCF-7 cells (Fig. 2D). Moreover, miR-638 overexpression also repressed cell migration while knockout of miR-638 promoted the migration of MCF-7 cells (Fig. 2E). Similarly, the same results of cell invasion regulated by miR-141-3p were also identified in MCF-7 cells (Fig. 2F). All these results suggested that miR-638 overexpression inhibited breast cancer progression.

### miR-638 can directly bind to HOXA9 in breast cancer

**Fig. 3:**
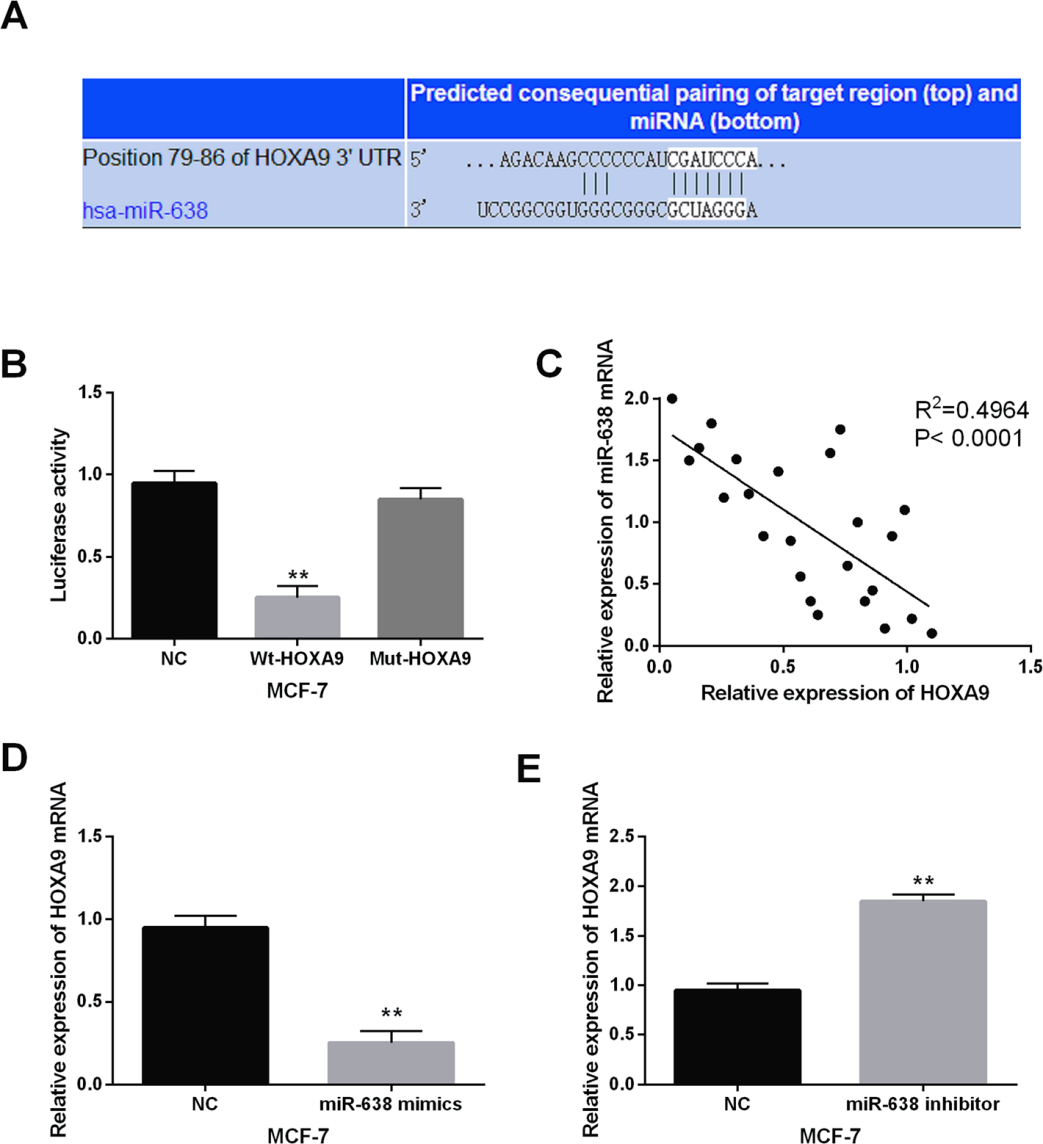
HOXA9 was a direct target of miR-638 in breast cancer. (**A**) HOXA9 had binding sites with miR-638. (**B**) Luciferase reporter assay. (C) miR-638 had negative correlation with HOXA9. (**D**, **E**) The expression of HOXA9 were observed in MCF-7 cells containing miR-638 mimics or inhibitor **P <0.01

The downstream target of miR-638 was identified in this study. HOXA9 was found to have binding sites with miR-638 predicted by TargetScan (http://www.targetscan.org/) (Fig. 3A). Then, luciferase reporter assay suggested that miR-638 mimics decreased the luciferase activity of Wt-HOXA9. But miR-638 mimics had little effect on that of Mut-HOXA9 (Fig. 3B). Moreover, HOXA9 expression was found to have negative correlation with miR-638 in breast cancer tissues (*R*^2^ = 0.4964, Fig. 3C). We also found that miR-638 mimics declined HOXA9 expression (Fig. 3D), and miR-638 inhibitor enhanced HOXA9 expression in MCF-7 cells (Fig. 3E). In a word, miR-638 can directly bind to HOXA9 and negatively regulate HOXA9 expression in breast cancer.

### HOXA9 was involved in breast cancer progression

**Fig. 4:**
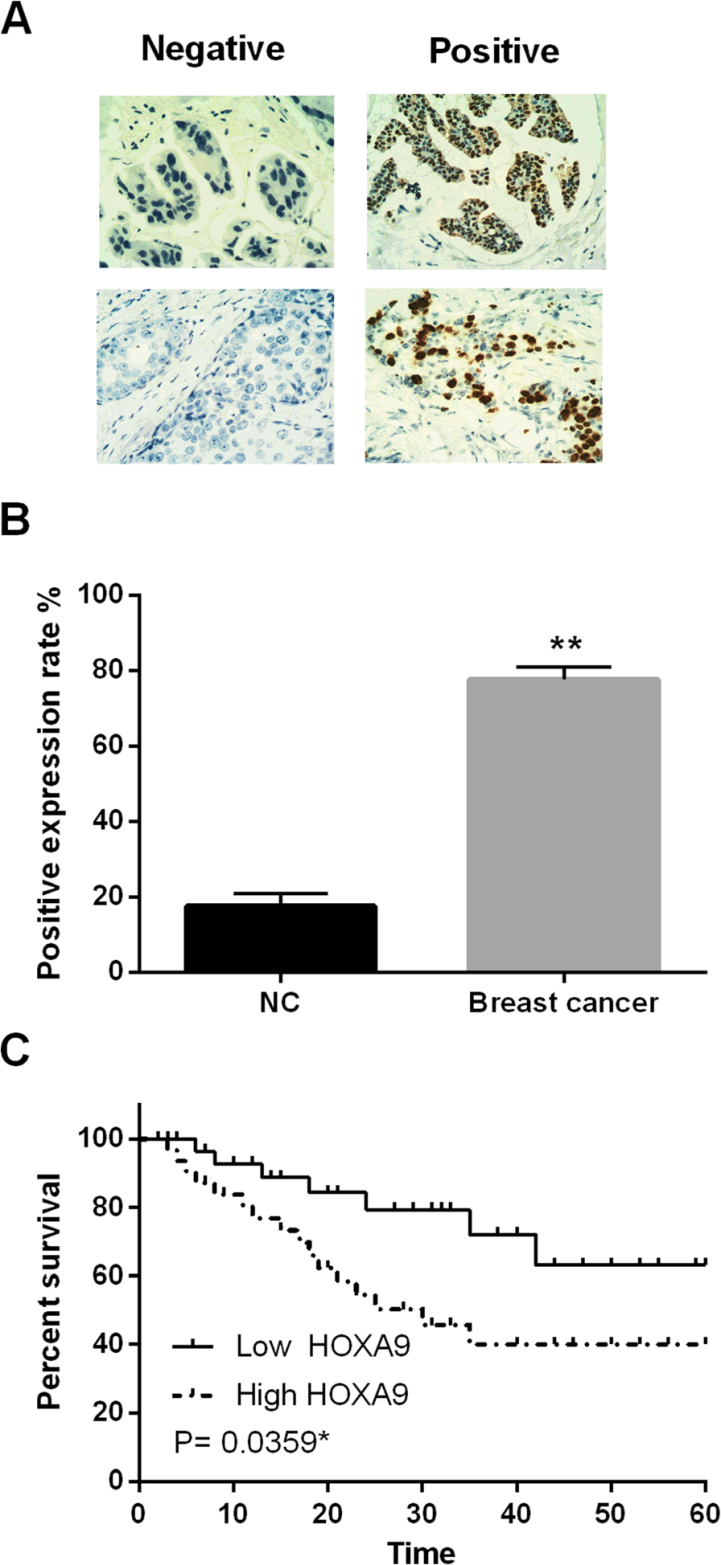
HOXA9 was upregulated in breast cancer tissues. (**A**, **B**) The protein expression of HOXA9 in breast cancer tissues detected by immunohistochemistry. (**C**) High HOXA9 expression was related to shorter overall survival of breast cancer patients. *P < 0.05

Next, HOXA9 expression was identified in breast cancer tissues. IHC showed that positive detection of HOXA9 protein expression was in the nucleus of breast cancer cells (Fig. 4A). Moreover, the upregulation of HOXA9 was detected in breast cancer tissues (Fig. 4B). Furthermore, breast cancer patients with high HOXA9 expression had shorter overall survival (Fig. 4C). According to these findings, HOXA9 was considered to participate in the pathogenesis and prognosis of breast cancer.

### miR-638 impeded the progression of breast cancer through targeting HOXA9

**Fig. 5:**
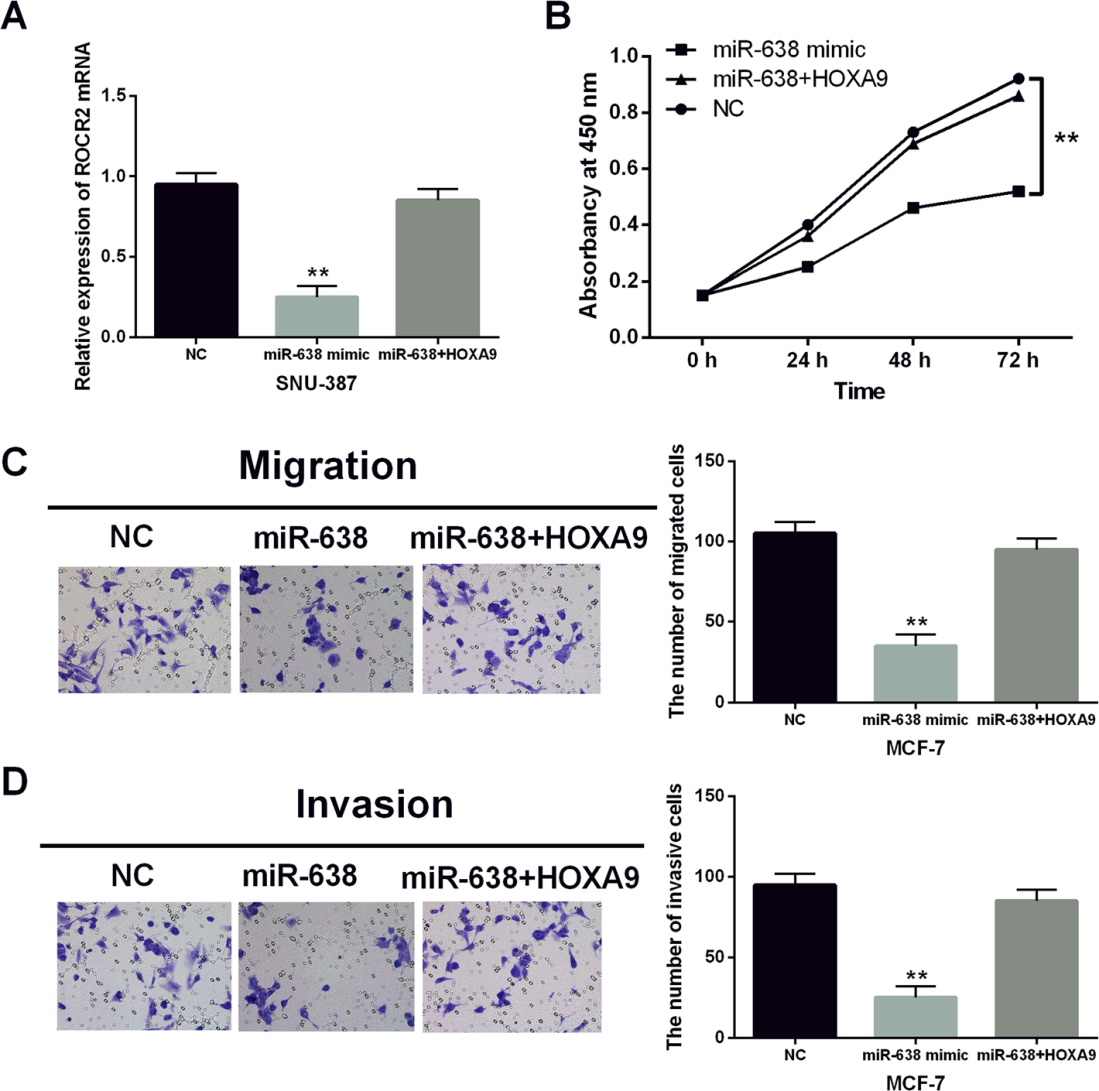
miR-638 impeded the progression of breast cancer through targeting HOXA9. (**A**) The expression of HOXA9 was measured in MCF-7 cells with HOXA9 vector and miR-638. (**B**) The cell proliferation was measured in MCF-7 cells with HOXA9 vector and miR-638. (**C**, **D**) The cell migration and invasion in MCF-7 cells with HOXA9 vector and miR-638 **P < 0.01

Then, miR-638 mimics and HOXA9 vector were transfected in MCF-7 cells to explore the relationship between miR-638 and HOXA9. The qRT-PCR assay suggested that the reduced HOXA9 expression induced by miR-638 mimics was recovered by HOXA9 vector in MCF-7 cells (Fig. 5A). Moreover, HOXA9 overexpression impaired the suppressive effect of miR-638 on MCF-7 cell proliferation (Fig. 5B). Consistently, the same results were also identified for cell migration (Fig. 5C) and invasion (Fig. 5D) in breast cancer. In brief, miR-638 was speculated to impede breast cancer progression by targeting HOXA9.

### miR-638 hindered EMT and Wnt/β-cadherin pathway in breast cancer

**Fig. 6:**
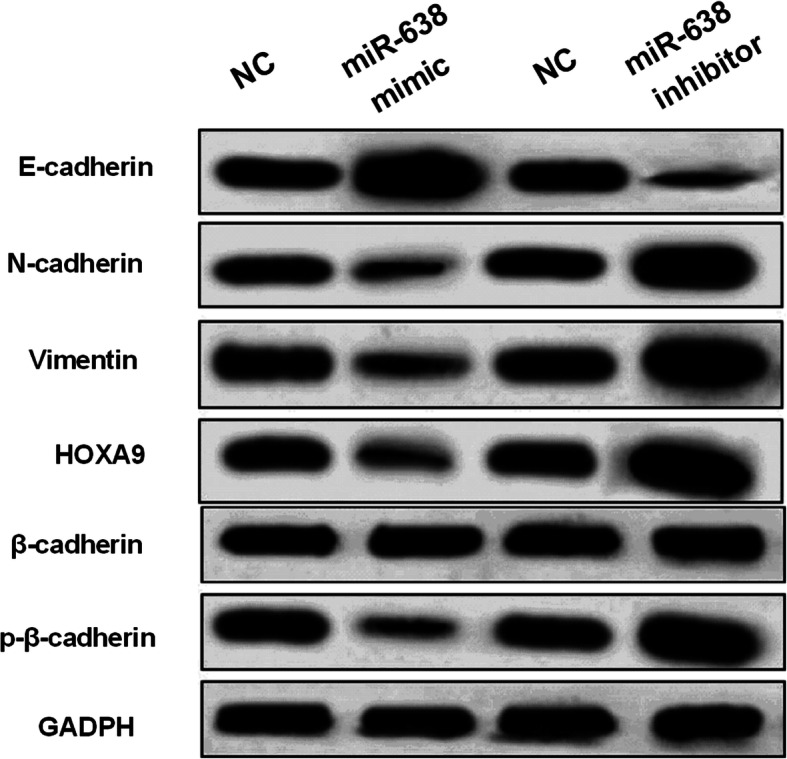
miR-638 hindered EMT and Wnt/β-cadherin pathway in breast cancer. Western blot analysis of E-cadherin, N-cadherin, Vimentin, β-cadherin, and p-β-cadherin in MCF-7 cells contained miR-638 mimics or inhibitor

Furthermore, we investigated the effect of miR-638 on EMT and Wnt/β-cadherin pathway. Upregulation of miR-638 was found to promote E-cadherin expression and inhibit N-cadherin and Vimentin expressions in MCF-7 cells (Fig. 6). And downregulation of miR-638 showed opposite results (Fig. 6). Moreover, miR-638 overexpression inhibited p-β-cadherin expression (Fig. 6), while miR-638 downregulation enhanced p-β-cadherin expression (Fig. 6). But β-cadherin expression was not affected by miR-638 in MCF-7 cells. Therefore, miR-638 may inhibit breast cancer tumorigenesis by hindering EMT and Wnt/β-cadherin pathway.

## Discussion

The global incidence of breast cancer has been on the rise since the late 1970s. Although China is not a country with high incidence of breast cancer, it should not be optimistic. In recent years, the growth rate of breast cancer incidence in China is 1-2% higher than that in countries with high incidence of breast cancer [25]. Therefore, people pay more and more attention to breast cancer. Recently, increasing miRNAs have been reported to participate in the pathogenesis of breast cancer, such as miR-22 [26] and miR-1271 [27]. In present study, downregulation of miR-638 was identified in breast cancer. And miR-638 exhibited suppressive effect on the development of breast cancer.

MiRNAs serve as important participants in regulating the behaviors of the cells, such as differentiation and proliferation. In recent 10 years, increasingly studies have indicated that miRNA dysfunction is one of the major causes leading the malignant progression of the tumors. In breast cancer, downregulated miR-143 is related with the poor prognosis of the patients with breast cancer, and miR-143 can effectively inhibit the malignant behaviors of the breast cancer cells via targeting HMGA2 [28]. In this study, miR-638 downregulation was related to worse prognosis in breast cancer patients. In previous studies, low miR-638 expression was found in hepatocellular carcinoma and related to its clinical significance [29]. Similarly, decreased miR-638 expression was also related to the clinic pathological characteristics in patients with breast cancer. Shi et al. proposed that decreased levels of miR-638 predicted poor prognosis in hepatocellular carcinoma [30]. In addition, miR-638 inhibited cell proliferation and invasion in human colorectal cancer [31], which is consistent with our results. Besides, miR-638 downregulation promoted EMT and cell invasion in colorectal carcinoma [32]. The same results were also identified in this study. Furthermore, it was observed that overexpression of miR-638 could hinder EMT and Wnt/β-cadherin pathways in breast cancer. Similarly, it was indicated that miR-638 inhibited the Wnt/β-catenin pathway in cervical cancer [33]. Furthermore, miR-638 directly binds to HOXA9 in breast cancer, and miR-638 impeded breast cancer progression through targeting HOXA9.

HOXA9 is a kind of homeoproteins which abnormally expressed aberrantly in human cancers [34]. Liu et al. proposed that HOXA9 was upregulated and functioned as an oncogene in chronic myeloid leukemogenesis [35]. In current research, we also found the upregulation of HOXA9 in breast cancer. Additionally, HOXA9 has been reported to be regulated by some miRNAs, such as miR-126 [36], miR-155 [37], and miR-196b [38]. Same as our findings, Wang et al. demonstrated that miR-133b suppressed metastasis by targeting HOXA9 in human colorectal cancer [39]. Zhang et al. indicated that miR-182 blocked Wnt/β-catenin signaling and suppressed cell proliferation in human osteosarcoma via targeting HOXA9 [40]. The dysfunction of Wnt/β-catenin pathway is involved in the formation and development of breast cancer. Jiang et al. have indicated that miR-449b-5p-mediated inactivation of Wnt/β-catenin pathway could effectively reduce the proliferation and invasion of the breast cancer cells [41]. In this study, miR-638 was found to suppress the tumorigenesis of breast cancer through targeting HOXA9 and suppressing Wnt/β-cadherin pathway. Therefore, this study suggested that miR-638 served as a tumor inhibitor to impede the malignant progression of breast cancer via suppressing the expression of HOXA9 and activation of Wnt/β-cadherin pathway.

## Conclusion

In conclusion, miR-638 downregulation was identified in breast cancer and related to adverse prognosis in breast cancer patient. Moreover, miR-638 inhibited breast cancer progression via targeting HOXA9 and suppressing EMT/Wnt/β-cadherin pathway. The findings may provide new thoughts for the diagnosis and therapies of breast cancer.

## Data Availability

The datasets used and/or analyzed during the current study are available from the corresponding author on reasonable request.
